# Inflammasomes in chronic liver disease: Hepatic injury, fibrosis progression and systemic inflammation

**DOI:** 10.1016/j.jhep.2024.06.016

**Published:** 2024-06-20

**Authors:** Vlad Taru, Gyongyi Szabo, Wajahat Mehal, Thomas Reiberger

**Affiliations:** 1 Division of Gastroenterology and Hepatology, Department of Medicine III, Medical University of Vienna, Vienna, Austria;; 2 Christian-Doppler Laboratory for Portal Hypertension and Liver Fibrosis, Medical University of Vienna, Vienna, Austria;; 3 Iuliu Hatieganu University of Medicine and Pharmacy, 4^th^ Dept. of Internal Medicine, Cluj-Napoca, Romania;; 4 Department of Medicine, Beth Israel Deaconess Medical Center and Harvard Medical School, Boston, MA, USA;; 5 Section of Digestive Diseases, Yale School of Medicine, New Haven, CT, USA;; 6 West Haven Veterans Medical Center, West Haven, CT, USA;; 7 Center for Molecular Medicine (CeMM) of the Austrian Academy of Science, Vienna, Austria

**Keywords:** inflammasome, toll-like receptor, nod-like receptor, hepatic stellate cell, Kupffer cell, macrophage, alcohol-related liver disease, metabolic dysfunction-associated steatotic liver disease, liver fibrosis, cirrhosis

## Abstract

Chronic liver disease leads to hepatocellular injury that triggers a pro-inflammatory state in several parenchymal and non-parenchymal hepatic cell types, ultimately resulting in liver fibrosis, cirrhosis, portal hypertension and liver failure. Thus, an improved understanding of inflammasomes – as key molecular drivers of liver injury – may result in the development of novel diagnostic or prognostic biomarkers and effective therapeutics. In liver disease, innate immune cells respond to hepatic insults by activating cell-intrinsic inflammasomes via toll-like receptors and NF-κB, and by releasing pro-inflammatory cytokines (such as IL-1β, IL-18, TNF-α and IL-6). Subsequently, cells of the adaptive immune system are recruited to fuel hepatic inflammation and hepatic parenchymal cells may undergo gasdermin D-mediated programmed cell death, termed pyroptosis. With liver disease progression, there is a shift towards a type 2 inflammatory response, which promotes tissue repair but also fibrogenesis. Inflammasome activation may also occur at extrahepatic sites, such as the white adipose tissue in MASH (metabolic dysfunction-associated steatohepatitis). In end-stage liver disease, flares of inflammation (*e.g*., in severe alcohol-related hepatitis) that spark on a dysfunctional immune system, contribute to inflammasome-mediated liver injury and potentially result in organ dysfunction/failure, as seen in ACLF (acute-on-chronic liver failure). This review provides an overview of current concepts regarding inflammasome activation in liver disease progression, with a focus on related biomarkers and therapeutic approaches that are being developed for patients with liver disease.

## Background

### Liver disease and inflammation

Liver fibrogenesis is a dynamic process characterised by excessive extracellular matrix deposition and reduced degradation by metalloproteinases, which occurs under chronic hepatic injury induced by different aggressors.^1^ The main cellular source of extracellular matrix is activated hepatic stellate cells (HSCs)^2^ that transdifferentiate into contractile, fibrogenic and pro-inflammatory myofibroblasts.^1,3^ While HSCs can be activated directly in various aetiologies of chronic liver disease, inflammatory cytokines and the inflammation-related cellular and mediator milieu is the key driving force of fibrosis progression in the liver.

Cirrhosis results from cumulative severe liver injury leading to extensive liver fibrosis, hepatic dysfunction and abnormalities in the sinusoidal vasculature.^1,4^ Recent clinical evidence shows that successful aetiologic treatment allows for fibrosis regression^5,6^ and regeneration of hepatic function.^7^ Systemic inflammation, indicated by elevated levels of interleukin (IL)-6, has been shown to increase with the progression of cirrhosis and to confer a higher risk of decompensation and mortality.^8^ Hence, understanding the molecular mechanisms that drive the hepatic pro-inflammatory environment will be crucial for the development of therapies to prevent severe complications and organ failure(s) and to promote liver disease regression.^9^

Inflammatory cells, including macrophages^10^ orchestrate hepatic inflammation and thereby contribute to progression of fibrosis and cirrhosis.^11^ The liver immune environment has been characterised by single-cell RNA sequencing experiments, which identified antigen-presenting cells (Kupffer cells, monocytes, liver sinusoidal endothelial cells and dendritic cells), natural killer (NK) cells, T cells, and B cells as resident cells in the healthy human liver.^12^ These cells play a key role in screening systemic and gut-derived antigens that reach the liver and classifying them as either harmless dietary and commensal compounds, or as pathogenic microbial elements.^13^ This balance between tolerance and immune response is profoundly altered in cirrhosis, resulting from the dysregulation and exhaustion of immune cells and reduced synthesis of key immune proteins.^13^

In this review, we aim to provide a translational overview of inflammasomes, which regulate the innate immune response, with a focus on their role in the initiation, progression and repair of liver disease.

### The inflammasome: molecular signalling complex

The factors that trigger innate inflammatory responses are classified into pathogen-associated molecular patterns (PAMPs) that are preserved components of infectious agents and damage (or danger)-associated molecular patterns (DAMPs) that are released during sterile cellular injury. Initial disruptions in the homeostatic immune environment induced by DAMPs/PAMPs prompt a type 1 inflammatory response, which is characterised by recruitment and activation of innate immune cells (*i.e*. monocytes and neutrophils) and production of pro-inflammatory cytokines, such as interferon-γ (IFN-γ), granulocyte-macrophage colony-stimulating factor, IL-6, tumour necrosis factor-α (TNF-α) and C–C motif chemokine ligand 2, which contribute to local tissue destruction.^14^ In order to restore the homeostatic balance, a reparative type 2 inflammatory response is mounted, involving a variety of inflammatory cells, such as M2 macrophages, type 2 innate lymphoid cells and NK cells, releasing type 2 cytokines (*i.e*. IL-4, IL-5 and IL-13).^15^ A switch from type 1 to type 2 responses is partially mediated by alarmins (*i.e*. IL-33) that are secreted following tissue injury.^16^

PAMPs and DAMPs are recognised by a considerable variety of pathogen recognition receptors. The pathogen recognition receptor family includes toll-like receptors (TLRs) and nucleotide-binding oligomerisation domain-like receptors (NLRs) expressed in different immune and parenchymal cells as membrane-bound and intracellular receptors, respectively.^17,18^ TLR activation commonly triggers myeloid differentiation factor 88 (MYD88)-dependent production of pro-inflammatory cytokines (TNF-α, IL-6 and IL-1). However, both MYD88-dependent and MYD88-independent TLR signalling pathways lead to phosphorylation (inactivation) of the inhibitory protein IκB, resulting in the release and translocation of NF-κB to the nucleus and transcription of inflammatory genes (including inflammasome components).^19,20^

Interactions between DAMPs/PAMPs, TLRs and NLRs determine the cytosolic assembly of supramolecular protein complexes, known as inflammasomes, which then activate caspase (CASP)1 and thereby promote pro-inflammatory signalling through IL-1β and IL-18.^21^ Subsequent release of IL-1β and IL-18 from inflammasome-activated cells induces a myriad of downstream events including IFN-γ secretion and NK cell recruitment and activation,^22^ inactivation of IL-33 and suppression of type 2 cytokine release,^23^ DNA fragmentation^24^ and secretion of pro-IL-1α, a tissue repair-promoting cytokine.^25^

Among the NLR family, inflammasomes remain the best characterised. Their structure contains three distinct domains: a C-terminal leucine-rich repeat that functions as a ligand-recognition domain for pathogens, an intermediate NOD (nucleotide-binding oligomerisation domain), also known as NACHT, which is important for self oligomerisation;^26^ and a variable N-terminal domain. NLRs that have been shown to form inflammasomes are: NLRP1, NLRP3, NLRP6, NLRP12, NLRP9b and NLRC4. In addition to the NLR family, other proteins contain a PYD domain and can form inflammasomes, such as absent in melanoma 2 (AIM2), interferon-inducible protein 16 and Pyrin.^27,28^

**Fig. 1.**
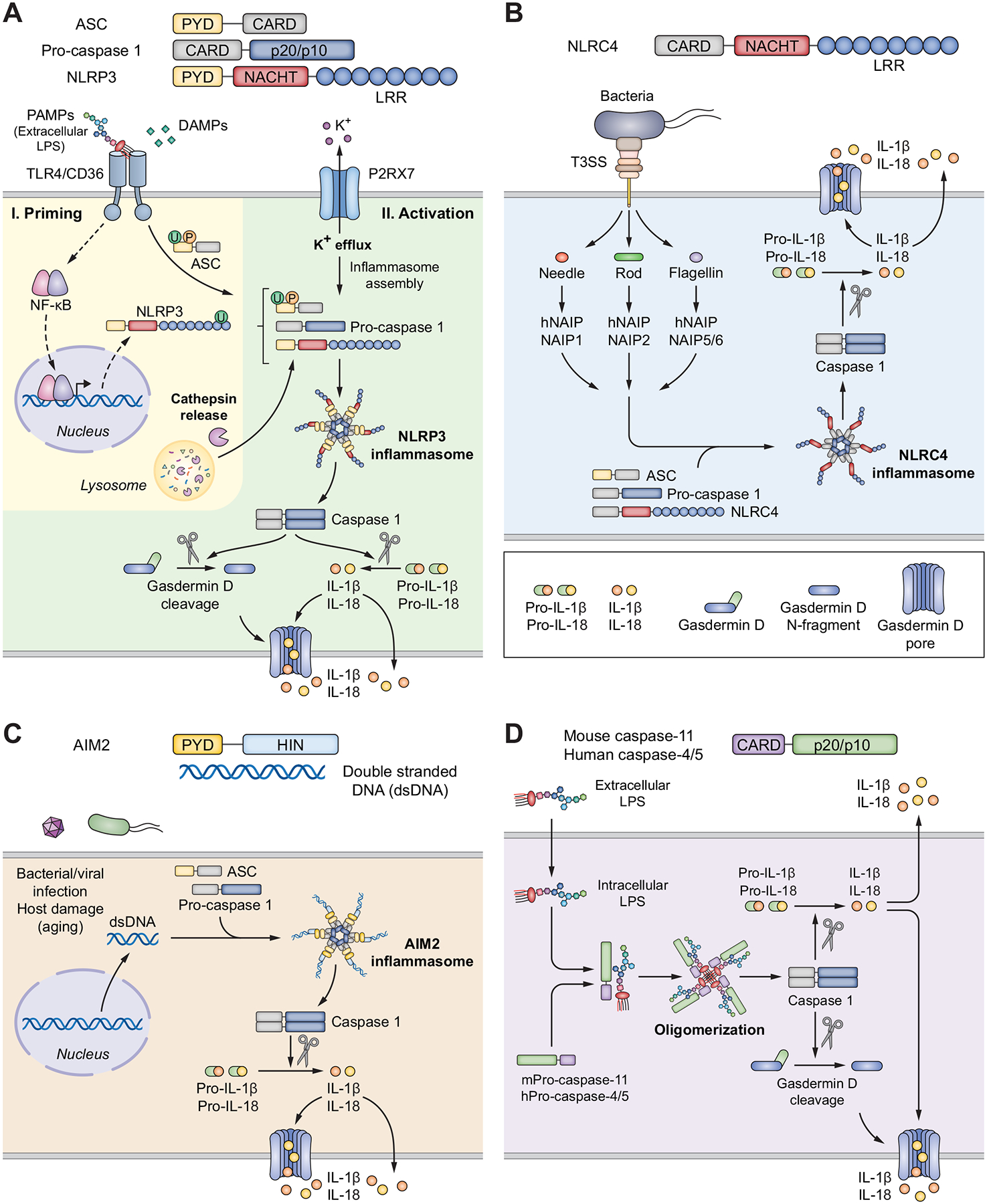
Mechanism of inflammasome assembly and activation. (A) NLRP3 inflammasome. PAMPs bind to the membrane TLR leading to the “priming” of the NLRP3 inflammasome by inducing de-ubiquitination in a non-transcriptional way^18,169^ and activating NF-κB signalling.^170^ The ubiquitination and phosphorylation of ASC^171^ completes the assembly of the NLRP3 inflammasome. The second step required for “activation” involves a second hit by a wide range of stimuli, such as extracellular ATP,^31^ urates,^34^ β-amyloid protein,^35^ and ROS,^36^ that subsequently result in intracellular “stress” events, such as excessive production of ROS, mitochondrial dysfunction, release of oxidized DNA, lysosomal dysfunction and cathepsin release, changes in intracellular calcium level, the formation of cell membrane pores, or potassium efflux.^172^ The mechanisms of NLRP3 activation involve the PYD domain of the NLRP3 protein which recruits the adaptor protein ASC, which then serves as a hinge for connecting the NLRP3 inflammasome complex to pro-CASP1 via the CARD. The assemble of NLRP3-ASC-proCASP1 complexes facilitates proximity-induced auto-processing and results in the formation of the active CASP1 enzyme, that produces IL-1β, IL-18 and GSDMD. The functional N-terminal GSDMD fragment binds to the cell membrane, oligomerises and creates a pore in the cell membrane causing ion gradient perturbations but also allowing for the release of selected molecules including mature IL-1β and IL-18.^173–175^ (B) NLRC4 inflammasome. Several bacterial components such as needle protein, rod or flagellin are sensed by the cell membrane complex T3SS. The ligand specificity is conferred by a range of receptors known as NAIPs, that connect the sensing signal of T3SS to the activation of NLRC4 inflammasome.^42^ From the seven genes that encode NAIPs, four are found in mice (NAIP1, NAIP2, NAIP5 and NAIP6) and only one in humans (hNAIP). The hNAIP senses all components of the T3SS, while each murine NAIP specifically binds to its cognate ligand.^176–178^ NLRC4 can directly recruit CASP1 through its CARD domain, so it may function in the absence of the ASC fragment.^179^ (C) AIM2 inflammasome. The AIM2 inflammasome is directly activated by bacterial or host defective double-stranded DNA (aging, activation of oncogenes, *etc*.). AIM2 lacks the CARD domain, so it requires ASC to recruit pro-caspase-1. The complex then oligomerises to form the AIM2 inflammasome, which can activate CASP1;^28^ (D) Non-canonical inflammasomes. The non-canonical activation of the inflammasome involves the presence of cytosolic LPS that recruits pro-CASP11 in mice and pro-CASP4 and pro-CASP5 in humans. The complex oligomerises and activates CASP11 (in mice) and CASP4/5 (in humans).^45^ The activated non-canonical caspases cannot directly process IL-1β and IL-18 but do so via alternative activation of the CASP1 enzyme. At the same time, the non-canonical caspases cleave GSDMD to its N-terminal active form, which leads to the formation of cell membrane pores and to a type of apoptotic cell death termed pyroptosis.^48,49^ ASC, apoptosis-associated speck-like protein containing a caspase recruitment domain; CARD, caspase recruitment domain; CASP, caspase; GSDMD, gasdermin D; IL-, interleukin-; LPS, lipopolysaccharide; NLRP3, Nod-like receptor pyrin domain containing-3; NAIPs, NLR family of apoptosis inhibitory proteins; PAMPs, pathogen-associated molecular patterns; ROS, reactive oxygen species; T3SS, type III secretion system; TLR, toll-like receptor.

The so called ‘canonical’ inflammasomes (including NLRP1, NLRP3, NLRC4, AIM2, IFl16, and Pyrin) all activate CASP1, as opposed to the “non-canonical inflammasomes” that activate CASP11 in mice and CASP4 and CASP5 in humans (Fig. 1). For an excellent overview of inflammasome cellular mechanisms in health and disease we recommend a recent review.^29^

#### Canonical inflammasomes

The NLRP1 was the first subset of NLRs that was shown to assemble, oligomerise and activate the CASP1 cascade.^21^ For a detailed description of the mechanisms of NLRP1 inflammasome assembly and activation, readers are referred to another excellent review.^30^

NLRP3 is currently the best characterised canonical inflammasome and was shown to be induced by a wide range of molecules, such as PAMPs derived from bacteria,^31^ viruses^32^ or fungi,^33^ or from host-derived factors (DAMPs) such as extracellular ATP,^31^ urates,^34^ β-amyloid protein,^35^ and reactive oxygen species (ROS).^36^ Despite extensive research, no unified mechanism for NLRP3 activation was agreed upon and many different theories have been proposed. The most accepted pathway is the “two-step” activation cascade (Fig. 1A).

The NLRC4/NAIP inflammasome is involved in host defence and plays a crucial role in the restriction of intra-epithelial bacterial colonisation during early stages of enterobacterial infection.^37,38^ The activation of NLRC4 is slightly different from the NLRP3, since this inflammasome mainly responds to bacterial flagellin and components of the bacterial type III secretory system^39,40^ (Fig. 1B).

Another class of inflammasomes do not contain NLRs, but instead contain pyrin and HIN200 domains (PYHIN) and are a family of IFN-induced proteins.^27^ From these, AIM2 was shown to activate CASP1. AIM2 binds directly to its ligand, which is double-stranded DNA^41^ and can detect both bacteria- and virus-derived cytosolic double-stranded DNA, as well as DNA from damaged cells^28,41–43^ (Fig. 1C).

The NLRP6 inflammasome has also been shown to be capable of activating CASP1, which is linked to homeostatic IL-18 secretion from intestinal epithelial cells and the maintenance of a healthy microbiota.^44^

#### Non-canonical inflammasome

Mouse CASP11 and human CASP4/5 can be directly activated in the host cell cytosol by lipopolysaccharide (LPS) released by Gram-negative bacteria^45–48^ (Fig. 1D). Interestingly, LPS directly binds to non-canonical caspases determining their oligomerisation and activation.^48^ CASP4/5 (human) and CASP11 (mouse) can also trigger pyroptosis in the absence of TLR stimulation and indirectly determine the secretion of mature cytokines by activating the assembly of the NLRP3 inflammasome and subsequently activating CASP1.^49–51^

Taken together these mechanisms underline the different host cell mechanisms for recognising DAMPs/PAMPs in the extracellular and intracellular space and responding via activation of canonical and non-canonical inflammasomes.

### Inflammasomes link innate and adaptive immunity

Three pathways are central to activation of the adaptive immune system: antigen receptor activation, co-stimulation by antigen-presenting cells and direct stimulation by inflammatory cytokines.^52^ Inflammasome activation can enhance each of these via inflammasome-mediated pyroptosis (releasing intracellular self and pathogenic antigens), IL-1β and IL-18 mediated upregulation of co-stimulatory signals on antigen-presenting cells, and direct activation of T and B cells.^53^ Inflammasome activation directly impacts CD4+ T cell activation, with IL-18 receptor activation driving T helper 1 CD4+ T cell induction and IL-1R activation driving T helper 17 CD4+ T cell induction, and both being important in anti-fungal responses.^54–56^ CD8+ T cells are also stimulated by inflammasome activation via IL-18, which mediates proliferation and survival of CD8+ T cells, and has been shown to be important in the response to *Salmonella*.^57^ Paradoxically in some systems, such as infection with *L. monocytogenes*, activation of inflammasome responses results in reduced CD8+ T cell responses, which may be due to increased pyroptotic death of key immune cells.^58^ Therefore, the clear ability of inflammasome activation to interact with the adaptive immune system likely has an impact on auto-inflammatory conditions, such as metabolic liver diseases, in which the adaptive immune system plays a significant role.^59,60^

## The inflammasome in liver disease

### Involvement of the inflammasome in liver fibrogenesis

The liver is exposed to many different microbial and viral signals that can potentially activate the innate immune system. On the one hand, hepatitis viruses directly elicit an immune response from the infected cells, while bacterial compounds which leak through the altered gut barrier and are carried to the liver via the portal vein maintain or further precipitate inflammation.^61^ On the other hand, damaged liver parenchymal and non-parenchymal cells release DAMPs that generate sterile signals that can activate immune cell receptors.^62^ These different mechanisms of inflammasome activation in liver diseases have been discussed in previous reviews.^63,64^

While inflammasomes were initially described and studied in cells of the innate immune system, such as macrophages or epithelial cells, where they are involved in maintaining barrier function,^65^ inflammasomes and their blueprint of activation markers were also identified in hepatic Kupffer cells (KCs) and to a lesser extent in HSCs and hepatocytes, during liver injury.^3^

Inflammasome activation was first linked to fibrosis development in hepatic tissue in human immortalized LX-2 cell lines and in stimulated primary mouse HSCs. The activation of the inflammasome in HSCs resulted in upregulation of the fibrogenic genes transforming growth factor-β (TGF-β) and collagen 1, together with a change in HSC morphology and chemotaxis.^66^ ROS release from dysfunctional mitochondria has also been demonstrated to occur in liver disease and is linked to activation of HSCs and increased liver fibrosis.^67,68^

**Fig. 2.**
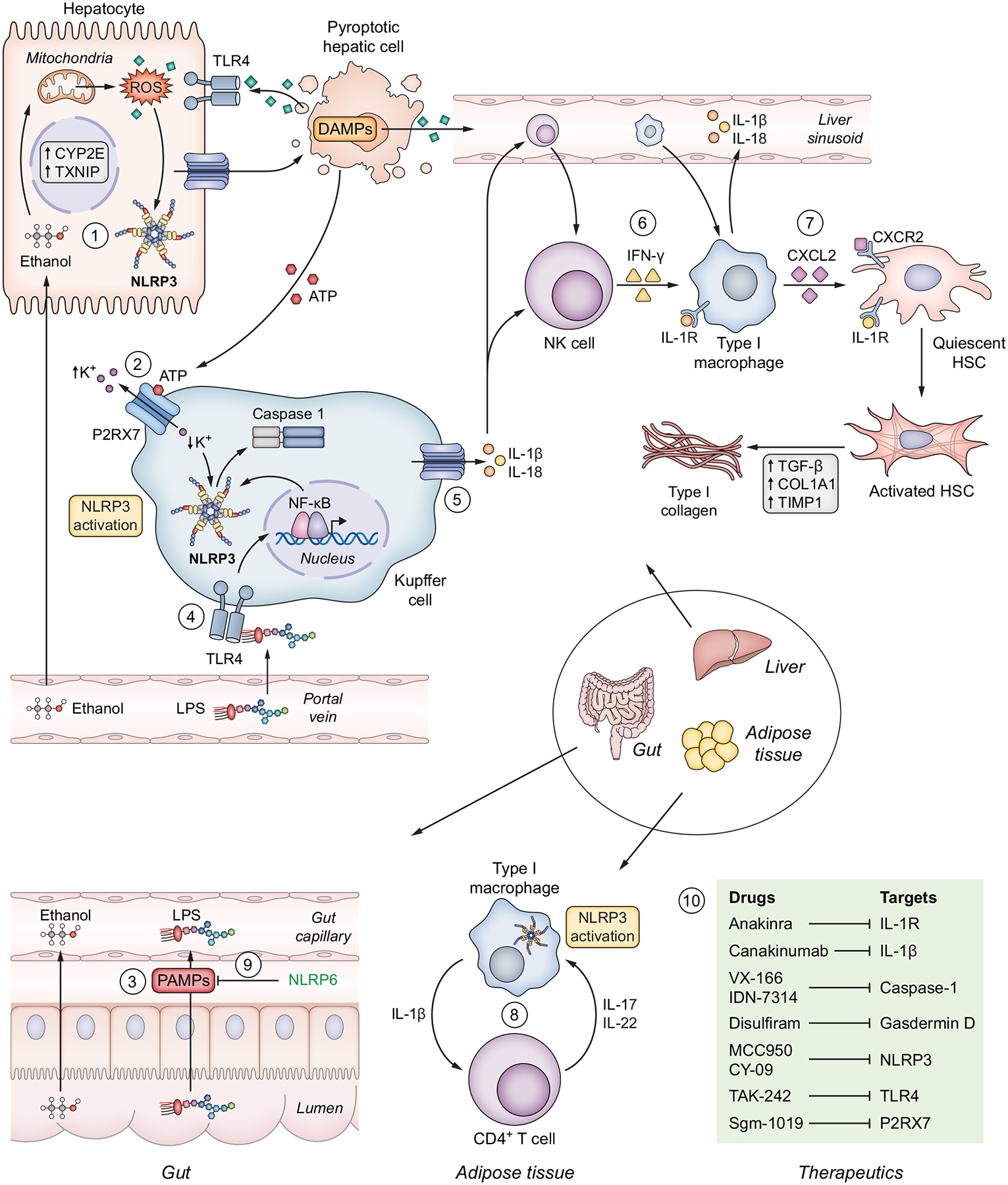
Crosstalk between resident liver cells and innate immune cells in ACLD. (1) In the early stages of ACLD, the central inflammatory stimuli (ROS, ATP, *etc*.) arise from damaged hepatocytes exposed to a causative (aetiological) factor such as ethanol, oxidized lipids, hepatitis viruses, etc.^95,96^ (2) Injured/damaged hepatocytes subsequently release molecules collectively known as DAMPs that can stimulate KCs and activate inflammasomes via the P2RX7 receptor (which senses extracellular ATP).^82^ (3) During late stages of ACLD, pathogens (*i.e*. LPS). leak through the damaged intestinal barrier into the portal venous circulation and finally reach the liver, (4) where they induce NLRP3 inflammasome priming via TLR4.^97^ (5) Release of pro-inflammatory cytokines through oligomerised gasdermin D pores in the cell membrane, first into the extracellular space and then into the liver sinusoid, determine (6) the recruitment of NK cells and BMDMs.^22,69^ The release of IFN-γ from NK cells leads to differentiation of BMDMs into type I macrophages, which further (7) activate quiescent hepatic stellate cells via the CXCL2/CXCR2 pathway, resulting in collagen production and extracellular matrix deposition and ultimately liver fibrosis.^73^ (8) Persistent inflammation of adipose tissue, characteristic of metabolic syndrome and MASH, is partially mediated by inflammasome activation in the infiltrative type I macrophages and interactions with CD4+ T lymphocytes.^121^ (9) The NLRP6 inflammasome displays a predominantly anti-inflammatory function, by maintaining the functionality of intestinal epithelium during alcohol-related injury.^94^ (10) Several therapeutic molecules have been developed that target components of the inflammasome pathway. ACLD, advanced chronic liver disease; BMDMs, bone marrow-derived macrophages; CXCL2, C-X-C motif chemokine ligand 2; CXCR2, CXCL2 receptor; DAMPs, damage-associated molecular patterns; HSC, hepatic stellate cell; IFN, interferon; KCs, Kupffer cells; LPS, lipopolysaccharide; MASH, metabolic dysfunction-associated steatohepatitis; NK, natural killer; NLRP3/6, Nod-like receptor pyrin domain containing-3/6; P2RX7, purinergic receptor 2X7; ROS, reactive oxygen species; TLR4, toll-like receptor 4.

In the context of liver injury, differential expression and activation of the NLRP3 inflammasome was investigated in hepatic cells (Fig. 2). Importantly, NK cells represent a major immune cell population that aggregates during liver injury (when TNF-α, granulocyte-macrophage colony-stimulating factor and IL-6 are released) and orchestrates a type 1 inflammatory response, which is associated with activation of the NLRP3 inflammasome and subsequent CASP1-mediated IL-1β secretion.^69^ Progressive tissue damage fuels chronic recruitment of IL-17+ neutrophils that activate HSCs, leading to secretion of TGF-β, collagen and extracellular matrix deposition and ultimately to fibrosis.^70^ While recruited bone marrow-derived macrophages generate a significant NLRP3-mediated response in the livers of mice and humans compared to other cell types,^71^ hepatocytes are also able to undergo CASP1-dependent pyroptosis and release inflammasome oligomers.^72^

Activation of HSCs plays a fundamental role in the development of liver fibrosis. HSCs can internalise extracellular NLRP3 inflammasome oligomers leading to fibrogenesis.^72^ During toxic liver injury, bone marrow-derived macrophages expressing MyD88 (a TLR4 adapter protein, indicating LPS responsiveness) infiltrate the liver and release CXCL2 (C-X-C motif chemokine ligand 2) that in turn activates quiescent HSCs to transdifferentiate into myofibroblasts, which is notably strongly linked to activation of the NLRP3 inflammasome. This fibrogenic response in HSCs was successfully abrogated by treatment with a CXCR2 (CXCL2 receptor) inhibitor, resulting in attenuated liver fibrosis *in vivo*.^73^

There is an abundance of experimental evidence that inflammasomes are involved in the pathophysiology of liver disease, especially, alcohol-related liver disease and alcohol-related steatohepatitis (ALD/ASH), metabolic dysfunction-associated steatotic liver disease/steatohepatitis (MASLD/MASH, formerly NAFLD/NASH), liver fibrosis and cirrhosis.^74^

### MASLD/MASH

#### Inflammasome activation in experimental MASH animal models

Several experimental models of MASH have been linked to inflammasome activation. Mice deficient in NLRP3 fed a high-fat diet (HFD) display increased liver steatosis, macrophage infiltration and liver injury, in parallel to increased adipose tissue inflammation, insulin resistance, alterations in gut microbiota composition and increased bacterial translocation.^75^ Consistently, CASP1, ASC and NLRP3-deficient mice fed a methionine-choline deficient diet had higher serum transaminases and increased liver steatosis compared to wild-type mice.^76^ The molecular mechanisms involved in the development of liver inflammation in inflammasome-deficient mice fed a methionine-choline deficient diet comprise intestinal dysbiosis, referred to as colitogenic microbiota, which seems to be mainly determined by inflammasome deficiency in the intestinal epithelial cells.^44,76^ Translocation of PAMPs to the portal circulation and subsequent hepatic TLR4 and TLR9 activation cause the intrahepatic release of TNF-α, which mediates hepatotoxic effects downstream of the altered microbiota.^76^ Additionally, increased steatosis observed in inflammasome knock-out models can be explained by upregulation of genes involved in lipid uptake and storage, increased oxidative stress, and a decreased antioxidant response/capacity.^75^

To explore NLRP3-mediated cell-to-cell communication, innovative animal models were developed that exhibited cell-specific knock-out of the *Nlrp3* gene in HSCs, hepatocytes and myeloid cells, respectively. Consequently, it was demonstrated that myeloid cells are predominantly responsible for mediating NLRP3 inflammasome activation in the liver, determining infiltration of neutrophils and directly promoting the profibrotic phenotype of HSCs, resulting in fibrosis progression.^77^ The same research group went on to demonstrate the role of IL-18 in mediating activation of HSCs and liver fibrosis. Moreover, treatment with an IL-18 antagonist reduced collagen deposition and increased metalloproteinase activity in myeloid-specific *Nlrp3* gain-of-function mutant mice.^78^

Another important activator of the inflammasome is extracellular ATP, which binds and activates the purinergic receptor 2X7 (P2RX7). Activation of P2RX7 has been associated with immune-related responses such as NLRP3 activation and IL-1β release.^79^ P2RX7 signalling emerged as a potential upstream cascade leading to NLRP3 activation in MASH and toxic murine models.^80–82^ *P2rx7*^−/−^ mice fed a HFD showed lower *Nlpr3, Casp1* and *Il1b* mRNA levels compared to controls.^82^ Similarly, significantly more inflammatory cells expressing *P2RX7* were present in liver samples from patients with MASH compared to controls.^80^

#### Lipid metabolism modulates inflammasome activation in MASH

The peroxisome proliferator-activated receptors (PPARs) are regarded as the master regulators of adipose tissue and liver metabolism. Perilipin 5 (*Plin5)*, a gene regulated by PPAR-α and induced during fasting, with roles in lipid peroxidation, was shown to mediate inflammasome activation in HFD-fed mice. Livers of *Plin5*^−/−^ mice displayed decreased lipid infiltration and inflammasome signalling molecules compared to wild-type when both groups were fed a HFD.^83^ Pan-activation of PPARs with the experimental drug IVA337 led to a decrease in *Nlrp3*, *Casp1* and *Nfkb* expression, reducing liver inflammation, fibrosis and even promoting reversion of liver fibrosis in comparison to single PPAR agonists.^84^

Sphingolipids are indispensable in cell membrane composition and are involved in a variety of cell processes, including inflammation, immunity and metabolism.^85^ Sphingomyelin synthetase 1 is a key enzyme that is overexpressed in metabolic dysfunction-induced liver injury and induces CASP1-dependent hepatic cell pyroptosis through the NLRC4 inflammasome pathway.^86^ Sphingosine 1-phosphate (S1P) and its receptor S1PR2, were incriminated as a factor in NLRP3 inflammasome activation, and silencing S1PR2 had promising anti-inflammatory and anti-fibrotic effects *in vivo*.^71^ Further research on the mechanism of S1P signalling revealed that S1P receptor 4 (S1PR4) was the most consistently overexpressed S1P receptor in the liver in different *in vivo* MASH models. Selectively inhibiting S1PR4 with the functional antagonist SLB736 ameliorated hepatic inflammation and fibrosis by inhibiting the NLRP3 inflammasome in different dietary models.^87^ Interestingly, continuous exposure to lipotoxic metabolites, such as oxidized free fatty acids, induces chronic endoplasmic reticulum (ER) stress, activation of the terminal unfolded protein response pathway and activation of the NLRP3 inflammasome, leading to hepatic inflammation and hepatic cell death.^88^ ER stress is generated by calcium transcription factors via the NLRP3 pathway (leading to disease progression), while blocking unfolded protein response-mediated ER stress can overcome these mechanisms.^89^

### ALD and ASH

#### Inflammasome activation in experimental ALD/ASH animal models

Numerous studies have reported on the pro-inflammatory effects of alcohol on hepatic cells. Activation of NLRP3 and CASP1, resulting in the release of IL-1β in serum, has been reported in ethanol-fed mice.^90^ In fact, in these *in vivo* models, expression of inflammasome components is increased in hepatic immune cells compared to parenchymal cells, while the knock-out of *Nlpr3*, *Casp1* or *Asc1* resulted in an abrogation of these mechanisms.^90^ The same group identified a role of hepatocyte-derived uric acid and ATP released upon alcohol-related liver injury, which act as a second stimuli to activate the NLRP3 inflammasome and determine the processing, activation and release of IL-1β, pointing to their role in inflammatory crosstalk between hepatocytes and hepatic immune cells. Furthermore, KCs were identified as the major liver-resident cell population involved in boosting inflammasome activation upon alcohol-related liver injury, releasing IL-1β and promoting the recruitment and activation of NK cells.^91,92^

Counterbalancing the pro-inflammatory effects of alcohol would prove quite challenging since human clinical trials with TNF-α antibodies were discontinued due to the increased incidence of infectious complications.^93^ On the other hand, targeting IL-1 signalling with an IL-1 receptor antagonist (IL-1Ra) in ethanol-fed mice led to a reduction in serum transaminases, liver steatosis and damage, and improved survival compared with saline-treated, alcohol-fed controls.^90^

Recently, a study investigated the role of the NLRP6 inflammasome in mediating the alterations of intestinal epithelium during alcohol-induced lived disease in a transgenic murine model. NLRP6-deficient mice presented hyperplastic goblet cells in the colonic epithelium, a decreased mucus layer, partially compromised intestinal barrier function and altered microbiota composition compared to wild-type alcohol-fed mice. Surprisingly, NLRP6 deficiency did not affect the degree of liver injury and steatosis, but strongly reduced the extent of hepatic immune cell recruitment.^94^

#### Receptor mediated and intracellular NLRP3 inflammasome signalling in ALD/ASH

Prolonged alcohol exposure induces CYP2E1 in hepatocytes, resulting in impairments of the oxidative cycle with excessive production of ROS and inducible nitric oxide synthase activation, ER stress and signalling through the TLR4/MyD88/NF-κB pathway.^95–97^ Intracellular mechanisms of NLRP3 inflammasome activation via ROS involve the thioredoxin-interacting protein (TXNIP), which directly interacts with the major antioxidant protein thioredoxin thereby inhibiting its antioxidant function.^98^ Ethanol-fed mice showed increased TXNIP mRNA and protein levels in the liver, while patients with ASH showed a modest increase in the *TXNIP* mRNA levels on liver biopsy.^99^

Another intracellular mechanism of regulation of NLRP3 activation involves spleen tyrosine kinase (SYK), which mediates the phosphorylation of ASC (apoptosis-associated speck-like protein containing a caspase recruitment domain) and thereby assembly of the NLRP3 inflammasome.^33^ Activated SYK levels were higher in the liver of ethanol-fed mice and patients with ASH compared to controls, and functional inhibition of SYK in murine ALD models resulted in decreased macrophage activation, IL-1β and CASP1 production, hepatic steatosis, injury and cell death.^100^

Knock-out of the gene *P2rx7*, which abrogated ATP-mediated activation of the NLRP3 inflammasome in ethanol-fed mice, yielded reduced serum levels of IL-1β and transaminases, and improved liver histology compared to controls. Moreover, transgenic mice overexpressing uricase, an enzyme that degrades uric acid, had significant protection from liver damage induced by ethanol exposure.^101^

### Inflammasome activity in end-stage liver disease

In cirrhosis and particularly in the setting of acute decompensation and acute-on-chronic liver failure (ACLF), a high-grade systemic inflammatory phenotype has been described both in experimental studies and in patients.^13,102^ Incipient studies identified higher circulating levels of IL-1β (as an indicator of inflammasome activation) in the context of acute alcohol-associated hepatitis (AH) and in patients with stable cirrhosis compared to no cirrhosis.^103,104^ Inflammasome activation was later identified in the livers of patients with AH and was linked to the formation of Mallory-Denk bodies.^105^ In AH, there is activation of non-canonical CASP11 (in mice) and CASP4 (in humans) but not of canonical CASP1/IL-1β, as opposed to a ‘chronic’ ALD mouse model and healthy controls, respectively. This leads to increased GSDMD, which was proven to have a causal effect on neutrophil infiltration and hepatocyte death.^106^ Recently, it was demonstrated that ASC protein specks accumulate in the liver of a murine model of AH and in the circulation of patients with AH. Interestingly, the ASC specks persist in the plasma and liver even after the binge episode, indicating that they likely play a role in the sustained inflammation present in AH^107^.

Signs of sterile inflammasome activation were identified in patients with decompensated cirrhosis with negative ascitic fluid and blood cultures. Levels of IL-18, but not IL-1β, were elevated in the serum of these patients compared to healthy controls, while AIM2 inflammasome activation via bacterial DNA led to an increase in IL-1β, IL-18 and activated CASP1 levels in peritoneal CD14+ macrophages isolated from ascitic fluid and correlated with disease severity assessed by Child-Pugh score.^108^ Importantly, IL-18 was an independent predictor of developing spontaneous bacterial peritonitis, indicating that increased sterile activation of the AIM2 inflammasome might be an early marker for disease progression in patients with decompensated cirrhosis and ascites. In another study, patients with compensated cirrhosis showed high levels of circulating IL-1α, while (previously) decompensated patients had higher levels of IL-1β.^109^

ACLF is a severe condition occurring in patients with cirrhosis, which is defined by hepatic and/or extrahepatic organ failures.^110^ Peripheral blood mononuclear cells isolated from patients with HBV-associated ACLF had higher expression of *ASC, NLRP3*, pro-*CASP1*, pro-*IL1B* mRNA, and higher serum protein levels of ASC and NLRP3 compared to patients with HBV without ACLF.^111^ In patients with HBV-ACLF, peripheral monocytes show a distinct pro-inflammatory phenotype and, importantly, their response to pathogens is impaired over time, suggesting progressive systemic immune paralysis.^112^

Collectively, these findings underline the importance of the inflammasome pathway in end-stage liver disease and in the superimposed acute injury (*i.e*. acute AH, infection, HBV flare, etc.), indicating that targeting inflammasome activation at specific levels may be especially beneficial in the context of “high-grade” systemic inflammation.^13^

## Inflammasome-mediated extrahepatic crosstalk in liver disease

There is a close functional relationship between adipose tissue and hepatic pathology in MASH. The effect of inflammasome activation in the white adipose tissue (WAT) is primarily related to a pro-inflammatory state and steatogenic response in the liver and this in turn is dependent on adipose tissue macrophages.^113^ Infiltration of adipose tissue with macrophages is closely related to the development of MASH, and conversely ablation of adipose macrophages partly reverses liver inflammation.^114–116^ Chronic excessive caloric intake results in the death of adipocytes which exposes adipose macrophages to a variety of stimuli, resulting in activation of inflammasome and other pro-inflammatory pathways.^117^ Interestingly, a temporal relationship between macrophage accumulation in the adipose tissue and the liver was described in a MASH murine model fed a high-fat high-cholesterol diet (HFC). CD11b+ and CD11c+ macrophages infiltrated the adipose tissue early, at 6 weeks post-HFC, while no infiltrates were present in the livers of HFC mice at this timepoint. Later, at 16 and 24 weeks post-HFC, there was a transition to increasing macrophage infiltrates in the liver and decreasing infiltrates in the adipose tissue, suggesting a switch from “simple” hepatic steatosis to inflammation (*i.e*. steatohepatitis) in the liver and development of MASH.^116^ Extensive gene profiling of different tissues showed that the differential regulation of several pro-inflammatory genes (*i.e. Il1b, Il6, Tnf*), as well as anti-inflammatory/pro-fibrogenic genes (*i.e. Il10, Tgfb1*), occurred sequentially in adipose tissue and then the liver parenchyma.^116^ The intersection of inflammasome biology with the role of adipose macrophages is demonstrated by increased expression of *NLRP3* and *IL1B* in the visceral fat of individuals with metabolic syndrome when compared to controls, and adipose tissue inflammation in mice has been shown to depend on CASP1.^118,119^ Conversely, following caloric restriction, the gene expression of *NLRP3* and *IL1B* is reduced in the subcutaneous fat of patients with insulin resistance.^120^ In addition to macrophage recruitment, there is also a phenotypic switch to an inflammatory phenotype, which depends on the NLPR3 inflammasome – as evident from experiments showing that genetic deletion of *NLRP3* results in downregulation of inflammatory macrophage genes such as TNF-α and C–C motif chemokine ligand 20.^120^ Inflammasome-mediated macrophage IL-1β production also initiates a positive feedback loop of IL-17 and IL-22 production by CD4+ T cells which further increases IL-1β production.^121^

In addition to tissue damage, adipose tissue macrophages with activated NLRP3 inflammasomes limit the ability of adipose tissue to adapt to metabolic stress. Specifically, treatment of adipocytes with IL-1β inhibits adipocyte differentiation, and inhibition of CASP1 increases the expression of adipogenesis genes.^122^ In metabolic syndrome, matrix remodelling in adipose tissue and adipose tissue fibrosis are increased. Inhibition of the NLRP3 inflammasome results in a reduction of matrix turnover and reduced adipose tissue fibrosis.^123^ In addition to the dominant WAT, mammals have brown adipose tissue (BAT) which is characterised by abundant mitochondria and a greater capacity to dissipate energy as heat. These adipose tissue populations are not entirely distinct, and WAT can convert to BAT with improved metabolic effects for the whole body.^124^ Increased expression of NLRP3-regulated inflammatory factors is associated with a reduction in BAT, and deletion of *Nlrp3* in mice results in greater conversion of WAT to BAT.^125^ Consistent with this, adipocytes treated with LPS-stimulated macrophage cytokines have reduced levels of genes known to be required for development of BAT.^126^

Collectively these data show that inflammasome components and function are key to many of the pathological changes in adipose tissues which occur in overnutrition, and which in turn drive MASH.

## Inflammasome-related biomarkers and therapeutics

### Biomarkers and surrogates reflecting NLRP3 inflammasome activation

Considering the complexity of the NLRP3 pathway and its molecular signalling nodes, it is important to assess both upstream and downstream molecular signalling in order to obtain a comprehensive understanding of NLRP3 involvement in liver disease. Biomarkers of the NLRP3 inflammasome pathway that reflect activation of different molecular components in the liver and/or blood samples for translational research are summarised in Table 1.

Currently, inflammasome biomarkers and surrogates are used in research but not for clinical decision making. However, they have allowed significant progress in the understanding of the contribution of the NLRP3 inflammasome in human liver disease. To this end, the upstream biomarker TLR4 protein was overexpressed in the liver biopsies of patients with decompensated *vs*. compensated cirrhosis,^127^ while *TLR4* gene expression did not differ in peripheral blood mononuclear cells between patients with cirrhosis *vs*. healthy controls.^128,129^

In patients with MASH, hepatic mRNA expression of *NLRP3, ASC, CASP1 and IL1B* were elevated compared to healthy controls.^130^ Moreover, patients with MASH showed a further increase in hepatic expression of *NLRP3*, *ASC*, *pro-CASP1*, *pro-IL1B* and *proIL18* as well as *IL1B* and *IL18*, compared to those with MASLD, and the expression of *pro*-*IL1B* moderately correlated with liver fibrosis.^131^ Similarly, serum levels of IL-18, but not IL-1β, were significantly higher in patients with MASLD compared to healthy controls.^132^ Both, serum non-cleaved GSDMD and N-terminal GSDMD protein levels progressively increased with the transition from MASLD to MASH and serum N-terminal GSDMD level showed good performance in distinguishing patients with MASH from those with MASLD.^133^

Patients with AH display higher serum IL-1β compared to healthy controls.^104^ On liver biopsy of patients with AH, activation of the non-canonical CASP-4/GSDMD pathway was demonstrated, with increased mature GSDMD levels compared to patients with ALD and healthy controls.^106^ Importantly, liver biopsies of patients with ALD cirrhosis showed increased expression of *CASP1*, *IL1B* and *IL18*, which also positively correlated with the severity of the histologic liver disease and the expression of other pro-inflammatory cytokines, chemokines and fibrotic genes.^134^

Interestingly, serum IL-1α and IL-1β levels seem to be of prognostic value as they predicted ACLF development in patients with compensated and decompensated cirrhosis, respectively.^109^ It remains to be studied if lower *vs*. higher NLRP3 inflammasome activation in the liver or in the systemic circulation correlates with subsequent fibrosis regression *vs*. stabilization/progression, and if inflammasome-related read-outs may serve as ‘dynamic’ biomarkers allowing for the prediction of liver-related events.

### Therapeutics targeting the inflammasome in liver disease

A thorough understanding of the inflammasome pathway paved the way for developing therapies that target inflammasome activation and up/downstream signalling. Next, we summarise the current pre-clinical research and results of previous studies, and current progress of clinical trials (Table 2). Inflammasome-targeted therapies specifically in the setting of ALD/MASLD have recently been reviewed.^135,136^

#### IL-1 inhibitors

IL-1β signals via its main receptor (IL-1R), which can be blocked by the IL-1R antagonist anakinra, an FDA-approved drug for treating rheumatoid arthritis. IL-1R blockade improved hepatic transaminase levels, liver histology and overall survival in murine models of ethanol-induced liver injury.^90,137^ Following these promising pre-clinical results, a randomised-controlled trial (RCT) investigating anakinra in combination with zinc and pentoxifylline in severe AH failed to demonstrate significant improvement in survival compared to treatment with prednisolone, although there was a tendency towards improved survival at 3 and 6 months.^138^ An ongoing RCT is investigating the benefit of treatment with canakinumab, a human monoclonal antibody against IL-1β in patients with AH (NCT03775109).

#### Caspase inhibitors

Treatment with pan-caspase inhibitors (VX-166 and IDN-7314) showed variable degrees of benefit in murine models of MASH.^139,140^ Emricasan, another pan-caspase inhibitor was evaluated in a RCT, including patients with decompensated MASH cirrhosis, which failed to show a benefit in terms of decompensation events and all-cause mortality.^139,141,142^ A selective CASP1 inhibitor reduced hepatic neutrophilic cell influx, TNF-α and hepatic fibrosis in a transgenicmurine MASH model.^143^

An important caveat – at least for long-term caspase treatment – is the inhibition of cell death, which may give rise to hepatocarcinogenesis and holds the risk of liver cancer development in patients with liver disease.^144^

#### GSDMD inhibitors

Necrosulfonamide, a necroptosis inhibitor that specifically blocks necrosis downstream of RIP3 (receptor-interacting serine-threonine kinase 3) reduced mortality in murine acute liver failure models.^145^ Interestingly, inhibiting ASK1 (apoptosis signal-regulation kinase 1) in NLRP3-mutant mice reduced liver fibrosis, hepatocellular death and hepatic pro-inflammatory TNF-α expression.^146^ However, clinical phase III trials failed to show benefits of the ASK1 inhibitor selonsertib in patients with MASH and bridging fibrosis or compensated cirrhosis.^147^ Similarly, selonsertib was evaluated in an RCT including patients with severe AH, where it failed to demonstrate any survival benefit compared to prednisolone treatment.^148^

#### NLRP3 inhibitors

The development of MCC950, a highly potent and selective small molecule inhibitor of the NLRP3 inflammasome^149^ enabled the exploration of targeted interventions for this particular pathway in liver disease. Initial investigation into the effect of MCC950 treatment in diet-induced/genetic murine models of MASH demonstrated beneficial effects on metabolism, inflammation, liver injury and fibrosis in the treatment groups *vs*. controls.^150^ Further investigations into the benefit of treatment with MCC950 were conducted *in vivo* in fulminant and acute hepatitis models,^151,152^ and in toxic and cholestatic chronic liver injury models,^153–155^ where it could alleviate liver injury and improve survival. Despite promising results, phase II clinical trials of MCC950 in patients with rheumatoid arthritis were halted due to liver toxicity related to treatment.^156^ Overall, the potential of MCC950 to specifically reduce liver inflammation and fibrosis seems promising, especially in the context of acute liver injury, but future clinical trials to investigate this drug in patients would need to be carefully designed with greater standardisation of the drug dose. Another direct NLRP3 inhibitor, CY-09, was evaluated alone or in combination with sleeve gastroplasty in MASLD mouse models.^157,158^ Treatment with CY-09 ameliorated obesity, insulin resistance and hepatic steatosis in mice fed a HFD.^157,158^ Further pre-clinical evaluation of CY-09 mechanisms in the setting of MASH seems warranted.

## Future perspective on inflammasome research

Hepatic and extrahepatic inflammation, liver fibrogenesis and regeneration represent dynamic processes with inflammasomes acting as friend-foe in their orchestration. In early stages of liver disease inflammasome activation protects the liver from pathogens and infection as well as from metabolic or oxidative stress by limiting PAMP/DAMP-related injury. With liver disease progression, chronic activation of inflammasomes leads to excessive release of pro-inflammatory cytokines, abundant immune cell recruitment and sustained activation of HSCs that all promote hepatic inflammation and fibrosis. Therefore, a detailed understanding of the inflammasome-mediated hepatic and extrahepatic intercellular crosstalk between immune and non-immune cells during liver injury is key to develop effective therapeutic strategies.

Thorough *in vivo* characterisation of key molecular nodes of the inflammasome pathways may allow for the identification of novel prognostic biomarkers and development of drugs to target fibrogenesis and excessive inflammation in patients with liver disease.

Previous trials have investigated the effects of targeting inflammasome downstream signals, such as IL-1R, IL-1β or pan-caspase blockade, but found limited efficacy. Novel approaches to directly inhibit GSDMD or NLRP3 in liver disease have not yet been investigated in clinical trials but represent promising therapeutic options. Inflammasome inhibition may be of particular relevance in patients with rapid progression of disease and a “high-grade” inflammatory phenotype, as clinically observed in severe AH or ACLF.

## Figures and Tables

**Table 1.** NLRP3 biomarkers and surrogates examined in different biological compartments of patients with chronic liver disease.

Biological compartment	Upstream		Priming					Activation			
	TLR4	NF-κB	NLRP3	ASC	Pro-CASP1	Pro-IL-1β	Pro-IL-18	CASP1	IL-1β	IL-18	GSDMD
Blood											
Protein level	-	-	✓	✓	-	-	-	✓	✓	✓	✓
			111,159	107,111				72,160	104	104,160	133
PBMCs											
Gene expression	✓	-	✓	✓	✓	✓	-		✓	✓	-
	128,129		111	107,111	111	111			128	161	
Protein expression	-	-	✓	✓	-	-	-	✓	✓	✓	-
			162	162				162	128,161	161	
Liver biopsy											
Gene expression	✓	✓	✓	✓	✓	✓	✓	✓	✓	✓	-
	127	163	130,131	130,131	131	131	131	130	130,131	130,131,134	
Protein expression	✓	✓	✓	✓	-	-	-	✓	✓	✓	✓
	127	164	130,131	107				165	165	165	106

ASC, apoptosis-associated speck-like protein containing a caspase recruitment domain; GSDMD, gasdermin D; NLRP3, Nod-like receptor pyrin domain containing-3; NF-κB, nuclear factor kappa B; PBMCs, peripheral blood mononuclear cells; pro-CASP1, pro-caspase-1; TLR-4, toll-like receptor 4.

**Table 2.** Pharmacological modulation of NLRP3 inflammasome in liver disease.

Mechanism of action	Drug	Pre-clinical studies		Clinical trials	
		Design	Results	Design	Results
IL-1R antagonist	Anakinra	Mice: WT; *Il1r-KO*; *Casp1-KO* and *Asc-KO* ethanol-rich/normal diet treated with anakinra/vehicle^90^	↓ Hepatic inflammation, ameliorated histology	RCT in severe AH: anakinra 100 mg/day SC (14 days) + pentoxyfilin 400 mg × 3/day (28 days) + zinc 220 mg/day (6 months) *vs*. methylprednisolone 32 mg/day^138^	No survival benefit at 28, 90 and 180 days
IL-1β antibody	Canakinumab			RCT in AH: canakinumab 3 mg/kg IV (day 1) *vs*. placebo^166^	Ongoing
Pan-CASP inhibitors	VX-166	Mice: obese; diabetic *(db/db)* and WT fed MCD/normal diet treated with VX-166/vehicle^140^	No effect on hepatic injury or liver cell death; ↓ pro-inflammatory cytokines and liver fibrosis	n.a.	n.a.
	Emricasan	Mice: WT fed HFD/normal diet treated with emricasan/vehicle^139^	↓ Hepatic inflammation, injury and fibrosis	RCT in NASH-related cirrhosis and severe portal hypertension (HVPG ≥12 mmHg): emricasan 5 mg, 25 mg, 50 mg *vs*. placebo for 48 weeks^141^	No significant differences in HVPG change or in clinical outcomes
				RCT in NASH-related decompensated cirrhosis: emricasan 5 mg or 25 mg ×2/day *vs*. placebo^142^	No differences in time-to-first decompensating event and all-cause mortality
CASP1 inhibitor	Ac-YVAD-cmk	Mice: *Ldlr-KO* fed LFD or HFD treated with Ac-YVAD-cmk/vehicle^143^	↓ Hepatic inflammation, steatosis and fibrosis	n.a.	n.a.
ASK1 inhibitor	GS-444217	Mice: *Nlrp3-KI* and WT fed a diet containing GS-444217/normal diet^146^	↓ Hepatic inflammation, fibrosis and hepatocellular death,	n.a.	n.a.
	Selonsertib	n.a.	n.a.	RCT in severe AH: selonsertib 18 mg + prednisolone 40 mg *vs*. placebo + prednisolone 40 mg/day for 4 weeks^148^	No significant improvement in liver function/survival
		n.a.	n.a.	RCT in NASH with bridging fibrosis F3/compensated cirrhosis F4: 2:2:1 for 18 mg, 6 mg or placebo for 48 weeks^167^	No significant improvement in fibrosis without worsening of NASH
NLRP3 inhibitor	MCC950	Mice: *A1ms1 (foz/foz)* and WT fed atherogenic/MCD/normal diet treated with MCC950/vehicle^150^	↓ Hepatic inflammation, fibrosis	n.a.	n.a.
		Mice: ALF model; WT treated with MCC950/vehicle followed by CCl_4_/vehicle injection^152^	↓ Hepatic inflammation, injury	n.a.	n.a.
		Mice: WT with BDL/control treated with MCC950/vehicle^155^	↓ Hepatic inflammation, injury, cholestasis, fibrosis	n.a.	n.a.
	CY-09	Mice: WT randomised to HFD/normal diet and to receive sleeve gastroplasty surgery/sham surgery and treated with CY-09/vehicle^157^	↓ Body weight, insulin resistance, hepatic steatosis	n.a.	n.a.
		Mice: WT fed HFD and randomised to CY-09/vehicle^158^	↓ Body weight, insulin resistance, hepatic steatosis	n.a.	n.a.
TLR4 inhibitor	TAK-242 (Resatorvid)	Rats: WT with BDL/control treated with TAK-242/vehicle followed by LPS/vehicle injection^168^	↓ Hepatic inflammation, injury and mortality	RCT in alcohol-related cirrhosis with AD/AH and ACLF grade 1 or 2 (NCT04620148)	Ongoing
		Rats: WT with BDL/control or GalN/vehicle followed by LPS/vehicle injection + prophylactic/therapeutic treatment with TAK-242/vehicle Mice: WT treated with CCl_4_/vehicle + prophylactic/therapeutic treatment with TAK-242/vehicle^127^	↓ Systemic and hepatic inflammation, ↓ hepatic and extrahepatic organ injury, ↓ mortality,	n.a.	n.a.
P2RX7 inhibitor	SGM-1019	Non-human primates: CCl_4_ treated/control + treatment with SGM-1019/OCA/control^80^	Ameliorated liver histology, ↓hepatic inflammation, injury and fibrosis	n.a.	n.a.

ACLF, acute-on-chronic liver failure; AD, acute decompensation; AH, alcohol-associated hepatitis; ALD, alcohol-associated liver disease; ALF, acute liver failure; BDL, bile duct ligation; CCl4, carbon-tetrachloride; GalN, d-galactosamine; HVPG, hepatic venous pressure gradient; IL-1R, interleukin 1 receptor; KI, knock-in; KO, knock-out; Ldlr, low-density lipoprotein receptor; LFD/HFD, low/high-fat diet; LPS, lipopolysaccharide; MCD, methionine-choline deficient diet; NASH, non-alcoholic steatohepatitis; P2RX7, purinergic receptor P2X7; OCA, obeticholic acid; RCT, randomised-controlled trial; TLR, toll-like receptor; WT, wild-type.

## Data Availability

This review article contains no original data.

## Key points

- A summary of key molecular mechanisms mediated by inflammasomes, linking innate and adaptive immune responses in liver disease progression, is provided.
- The molecular crosstalk between extrahepatic inflammasome signalling and hepatic responses are reviewed.
- Inflammasome-related biomarker development and their mechanistic relevance in liver disease are summarized.
- Potential therapeutics targeting inflammasome signalling to ameliorate liver disease progression are discussed.

## Abbreviations

ACLF: acute-on-chronic liver failure
AH: alcohol-related hepatitis
AIM2: absent in melanoma 2
ALD: alcohol-related liver disease
ASC: apoptosis-associated speck-like protein containing a caspase recruitment domain
ASH: alcohol-related steatohepatitis
ASK1: apoptosis signal-regulation kinase 1
BAT: brown adipose tissue
CASP: caspase
DAMPs: damage-associated molecular patterns
ER: endoplasmic reticulum
GSDMD: gasdermin D
HFC: high-fat high-cholesterol diet
HFD: high-fat diet
HSCs: hepatic stellate cells
IFI16: interferon-inducible protein 16
IFN-γ: interferon-γ
IL-: interleukin
IL-1Ra: IL-1 receptor antagonist
KCs: Kupffer cells
LPS: lipopolysaccharide
MASH: metabolic dysfunction-associated steatohepatitis
MASLD: metabolic dysfunction-associated steatotic liver disease
MYD88: myeloid differentiation factor 88
NK: natural killer
NLRP3: nod-like receptor pyrin domain containing-3
NLRs: nucleotide-binding oligomerisation domain-like receptors
P2RX7: purinergic receptor 2X7
PAMPs: pathogen-associated molecular patterns
PLIN5: perilipin 5
PPARs: peroxisome proliferator-activated receptors
ROS: reactive oxygen species
S1P: sphingosine 1-phosphate
SYK: spleen tyrosine kinase
TGF-β: transforming growth factor β
TLRs: toll-like receptors
TNF-α: tumour necrosis factor α
TXNIP: thioredoxin-interacting protein
WAT: white adipose tissue
